# Community Experience of COVID-19 Hospitalizations

**DOI:** 10.7759/cureus.116652

**Published:** 2026-09-21

**Authors:** Kourosh Safai, Alexis Case, Amanda Frugoli, Timothy Pisarski, Kaylie Heyer, Graal Diaz

**Affiliations:** 1 Graduate Medical Education, Community Memorial Healthcare, Ventura, USA; 2 Pacific Inpatient Physicians, Community Memorial Hospital, Ventura, USA

**Keywords:** acute respiratory distress syndrome (ards), ards, covid, covid 19, covid-19 pandemic, covid-induced ards, pandemic

## Abstract

Background

Every American was affected by the COVID-19 pandemic. The novelty and virulence spurred the rapid evolution of treatment strategies. While large multicenter studies established key mortality risk factors and treatment efficacy, community hospital experiences are underrepresented.

Methods

This retrospective study examines clinical characteristics, mortality predictors, and outcome trends among 1,949 adults hospitalized with COVID-19 at a Southern California community hospital from January 31, 2020, through May 11, 2023.

Results

Overall mortality was 7.6%, and the mean length of stay was 9.2 days. Older age was independently associated with increased mortality, with the odds of death nearly doubling for every decade of life (adjusted odds ratio (aOR) 1.98, 95% CI 1.65-2.39, P < 0.001). Univariable laboratory predictors of inpatient mortality, and log transformation of D-dimer, demonstrated that D-dimer was the best predictor of inpatient mortality. Septic shock was associated with a twofold increase in mortality (aOR 2.26, 95% CI 1.11-4.63, P = 0.025). Respiratory failure requiring a mechanical ventilator is a marker for disease severity and was the strongest predictor of mortality in this cohort. Mechanical ventilation was associated with a 10-fold increase in the odds of death (aOR 10.07, 95% CI 6.18-16.38, P < 0.001). Multivariable analysis comparing mortality and pharmaceutical treatments identified that receipt of systemic corticosteroids was associated with higher odds of in-hospital mortality (aOR 6.70; 95% CI 4.00-11.22; P < 0.001). Receipt of tocilizumab was also associated with higher mortality (aOR 3.87; 95% CI 2.06-7.26; P < 0.001). Time-lapse analysis of the interval between admission and mortality demonstrated a 23% reduction in the odds of death per successive year (aOR 0.77 per year, 95% CI 0.62-0.96, P = 0.018).

Conclusion

Mortality decreased nearly one-fourth each year. D-dimer was the best laboratory predictor of death. Older age, septic shock, and ventilator need were associated with increased risk of inpatient mortality. Receipt of systemic steroids and tocilizumab was associated with higher mortality. These are not causal treatment effects, as medication receipt at that time was related to disease severity. These community hospital findings complement larger studies and reinforce the importance of early risk stratification and evidence-based treatments.

## Introduction

The SARS-CoV-2 pandemic or COVID-19 caused substantial morbidity and mortality worldwide, with more than one million deaths in the United States alone [1-3]. Beyond straining healthcare systems, the pandemic prompted rapid advances in clinical research and treatment development [4]. Prior studies identified obesity, ethnicity, and social determinants of health as important predictors of hospitalization and mortality. Laboratory evaluations identified D-dimer and monocyte distribution width (MDW) as having the highest association with disease severity and death [5-12].

Due to the novelty, experimental treatment interventions were tested simultaneously, with adoption and abandonment in real time. Early interventions such as convalescent plasma showed initial promise but ultimately did not demonstrate a clear mortality benefit in larger trials [13,14]. In contrast, remdesivir became the first FDA-approved antiviral for COVID-19 and was associated with shorter recovery time and improved outcomes in selected hospitalized patients [15-21]. For severe disease, immunomodulatory therapies became central to management. Dexamethasone demonstrated the strongest evidence for reducing mortality in patients requiring supplemental oxygen or ventilatory support and became guideline-directed therapy, whereas other corticosteroids showed less consistent benefit [22-25]. Interleukin inhibitors such as tocilizumab were also evaluated for severe inflammatory lung disease, with mixed results across randomized trials [26,27].

Despite extensive literature on COVID-19, few studies have examined the full course of the pandemic from the perspective of community hospitals or provided a summative description throughout the pandemic. In this study, we describe a community hospital's experience across the pandemic, focusing on clinical characteristics, treatment patterns, predictors of mortality, and outcome trends among adults hospitalized with COVID-19. We also evaluate temporal changes in hospital mortality with time-lapse analysis.

## Materials and methods

This retrospective study aimed to characterize the clinical features, treatment patterns, predictors of in-hospital mortality, and temporal trends in outcomes among adults hospitalized with COVID-19 at Community Memorial Healthcare in Ventura, CA, USA, between January 2020 and May 2023. A secondary objective was to evaluate changes in mortality over successive pandemic years as clinical management evolved. Study dates correlate with the Health and Human Services (HHS) pandemic declaration. The cohort was generated using ICD-10 code U07.1 and matched to positive COVID-19 results. Patients tested for isolation purposes or those with asymptomatic positive test results were not included.

In-hospital mortality was determined by discharge disposition, with patients coded as “Expired 20” classified as having died during hospitalization. Baseline demographic variables included age, sex assigned at birth or gender, insurance status, race and ethnicity, and BMI. Receipt of convalescent plasma, azithromycin, doxycycline, remdesivir, tocilizumab, and ventilator use were dichotomous variables.

Continuous variables were summarized as mean (SD), and categorical variables were summarized as No. (%). Between-year comparisons used one-way ANOVA for continuous variables and χ² tests for categorical variables.

Due to the retrospective study design, COVID-19 treatments with convalescent plasma and pharmaceutical treatments reflect physicians' discretion and the community standard of care at the time. One thousand nine hundred forty-nine patients were included in the multivariable analysis comparing treatments and inpatient mortality. To improve the area under the curve (AUC) fit, age and sex were required.

Mortality trends by year used a linear probability model; length-of-stay trends used linear regression. Mortality predictors were evaluated using logistic regression. Laboratory predictors were log2-transformed and standardized before modeling because of right-skewed distributions. Analyses involving laboratory tests were limited by missingness, particularly for ferritin, D-dimer, and sedimentation rate (SEDRT). The following statistical analyses were applied using open-source R statistical software packages (https://www.r-project.org).

## Results

A total of 1,949 COVID-19-related hospital admissions were analyzed across admission years 2020 through 2023. Overall mortality was 7.6%, and the mean length of stay was 9.2 days. Table 1 summarizes patient characteristics.

**Table 1:** Patient characteristics by admission year. Values are mean (SD) for continuous variables and No. (%) for categorical variables. P-values compare admission years using one-way ANOVA for continuous variables and χ² tests for categorical variables. N: total number; Y: yes; MCAL: MediCal or Gold Coast insurance; MCAR: Medicare insurance; Y0: year 0, time 0; LOS: length of stay; SIRS: systemic inflammatory response syndrome; SEDRT: sedimentation rate

Characteristic	Overall	2020	2021	2022	2023	P-value
Age at visit	67.2 (18.2)	63.5 (18.7)	64.3 (17.8)	68.4 (18.0)	71.3 (17.5)	<0.001
LOS (days)	9.2 (33.4)	8.9 (8.7)	9.8 (19.6)	6.8 (20.5)	12.9 (64.4)	0.062
ICU days	7.0 (9.2)	7.8 (7.8)	9.6 (11.7)	5.3 (7.4)	4.9 (7.9)	<0.001
Pat_BMI	28.8 (7.7)	30.0 (8.6)	30.1 (7.7)	28.3 (7.5)	27.4 (7.1)	<0.001
Ferritin	624.5 (867.7)	782.0 (1,036.2)	677.8 (819.6)	482.0 (853.9)	351.3 (481.1)	0.006
D-dimer	3,919.4 (12,796.9)	2,033.8 (4,482.7)	5,136.6 (16,409.3)	3,688.3 (10,978.4)	2,139.6 (2,430.5)	0.05
Procalcitonin	2.4 (24.6)	0.4 (0.7)	2.0 (14.1)	3.6 (38.1)	4.6 (30.5)	0.382
Lactate	1.7 (1.2)	1.7 (1.4)	1.7 (1.1)	1.7 (1.2)	1.8 (1.3)	0.922
CRP	9.8 (8.2)	10.2 (7.6)	10.7 (8.1)	8.6 (7.7)	9.5 (9.8)	0.007
SEDRT	54.4 (36.0)	67.3 (36.1)	57.7 (35.7)	47.2 (35.2)	53.0 (35.1)	0.002
Azithromycin or doxycycline						
N	1,402 (71.9)	126 (45.0)	270 (54.4)	652 (84.8)	354 (87.6)	<0.001
Y	547 (28.1)	154 (55.0)	226 (45.6)	117 (15.2)	50 (12.4)	
Actemra (tocilizumab)						
N	1,881 (96.5)	265 (94.6)	451 (90.9)	763 (99.2)	402 (99.5)	<0.001
Y	68 (3.5)	15 (5.4)	45 (9.1)	6 (0.8)	2 (0.5)	
Systemic steroids (oral and IV)						
N	942 (48.3)	89 (31.8)	121 (24.4)	467 (60.7)	265 (65.6)	<0.001
Y	1,007 (51.7)	191 (68.2)	375 (75.6)	302 (39.3)	139 (34.4)	
Remdesivir (Veklury)						
N	1,393 (71.5)	187 (66.8)	301 (60.7)	603 (78.4)	302 (74.8)	<0.001
Y	556 (28.5)	93 (33.2)	195 (39.3)	166 (21.6)	102 (25.2)	
Convalescent plasma						
N	1,905 (97.7)	255 (91.1)	477 (96.2)	769 (100)	404 (100)	<0.001
Y	44 (2.3)	25 (8.9)	19 (3.8)	0 (0.0)	0 (0.0)	
FinancialClassID						
Commercial	644 (33)	98 (35)	161 (32.5)	259 (33.7)	126 (31)	
MCAL	665 (34.1)	104 (37.1)	196 (39.5)	236 (30.7)	129 (31.9)	
MCAR	633 (32.5)	78 (27.9)	137 (27.6)	272 (35.4)	146 (36.1)	
Self	7 (0.4)	0 (0.0)	2 (0.4)	2 (0.3)	3 (0.7)	
Sex						
Female	930 (47.7)	122 (43.6)	227 (45.8)	373 (48.5)	208 (51.5)	0.159
Male	1,019 (52.3)	158 (56.4)	269 (54.2)	396 (51.5)	196 (48.5)	
Ethnicity_name						
American Indian or Alaska Native	2 (0.1)	0 (0.0)	0 (0.0)	1 (0.1)	1 (0.2)	<0.001
Asian	55 (2.8)	7 (2.5)	17 (3.4)	17 (2.2)	14 (3.5)	
Black or African American	40 (2.1)	5 (1.8)	8 (1.6)	21 (2.7)	6 (1.5)	
Hispanic	794 (40.8)	155 (55.4)	254 (51.2)	256 (33.3)	129 (32.0)	
White	1,046 (53.7)	112 (40.0)	215 (43.3)	467 (60.7)	252 (62.5)	
Died						
N	1,801 (92.4)	259 (92.5)	427 (86.1)	732 (95.2)	383 (94.8)	<0.001
Y	148 (7.6)	21 (7.5)	69 (13.9)	37 (4.8)	21 (5.2)	
ICU admission						
N	1,540 (79.0)	192 (68.6)	375 (75.6)	653 (84.9)	320 (79.2)	<0.001
Y	403 (20.7)	86 (30.7)	121 (24.4)	114 (14.8)	82 (20.3)	
Y0	6 (0.3)	2 (0.7)	0 (0.0)	2 (0.3)	2 (0.5)	
Ventilator						
N	1,640 (84.1)	229 (81.8)	388 (78.2)	672 (87.4)	351 (86.9)	<0.001
Y	309 (15.9)	51 (18.2)	108 (21.8)	97 (12.6)	53 (13.1)	
COVID-19 Dx						
N	190 (9.7)	12 (4.3)	15 (3.0)	68 (8.8)	95 (23.5)	<0.001
Y	1,759 (90.3)	268 (95.7)	481 (97.0)	701 (91.2)	309 (76.5)	
SIRS						
N	1,929 (99.0)	278 (99.3)	491 (99.0)	763 (99.2)	397 (98.3)	0.438
Y	20 (1.0)	2 (0.7)	5 (1.0)	6 (0.8)	7 (1.7)	
Sepsis						
N	1,677 (86.0)	214 (76.4)	406 (81.9)	704 (91.5)	353 (87.4)	<0.001
Y	272 (14.0)	66 (23.6)	90 (18.1)	65 (8.5)	51 (12.6)	
Severe sepsis						
N	1,745 (89.5)	221 (78.9)	421 (84.9)	731 (95.1)	372 (92.1)	<0.001
Y	204 (10.5)	59 (21.1)	75 (15.1)	38 (4.9)	32 (7.9)	
Septic shock						
N	1,865 (95.7)	258 (92.1)	473 (95.4)	747 (97.1)	387 (95.8)	0.006
Y	84 (4.3)	22 (7.9)	23 (4.6)	22 (2.9)	17 (4.2)	

For laboratory predictors, univariable models showed that CRP, lactate, D-dimer, ferritin, and procalcitonin were associated with mortality (Table 2). In the complete-case adjusted model including all six laboratory tests, no individual laboratory test remained statistically significant, likely reflecting high missingness or blank data fields and reduced sample size (Figure 1).

**Table 2:** Laboratory predictors of in-hospital mortality. Odds ratios (ORs) are per 1-SD increase in log2-transformed laboratory value. The adjusted model includes ferritin, D-dimer, procalcitonin, lactate, CRP, and SEDRT. Complete-case N = 110; deaths = 24; C statistic = 0.682. SEDRT: sedimentation rate

Rank	Lab test	Univariable N	Deaths	Univariable OR (95% CI)	Univariable P-value	Adjusted OR (95% CI)	Adjusted P-value
1	Procalcitonin	969	114	1.19 (1.01-1.39)	0.034	0.59 (0.31-1.13)	0.11
2	CRP	1,015	112	1.83 (1.45-2.31)	<0.001	1.9 (0.83-4.34)	0.13
3	Lactate	1,063	124	1.6 (1.36-1.88)	<0.001	1.45 (0.82-2.56)	0.2
4	SEDRT	416	43	1.27 (0.89-1.82)	0.184	0.81 (0.41-1.62)	0.551
5	D-dimer	761	110	1.57 (1.31-1.87)	<0.001	1.11 (0.72-1.7)	0.635
6	Ferritin	529	78	1.66 (1.27-2.17)	<0.001	1.06 (0.59-1.91)	0.842

**Figure 1:**
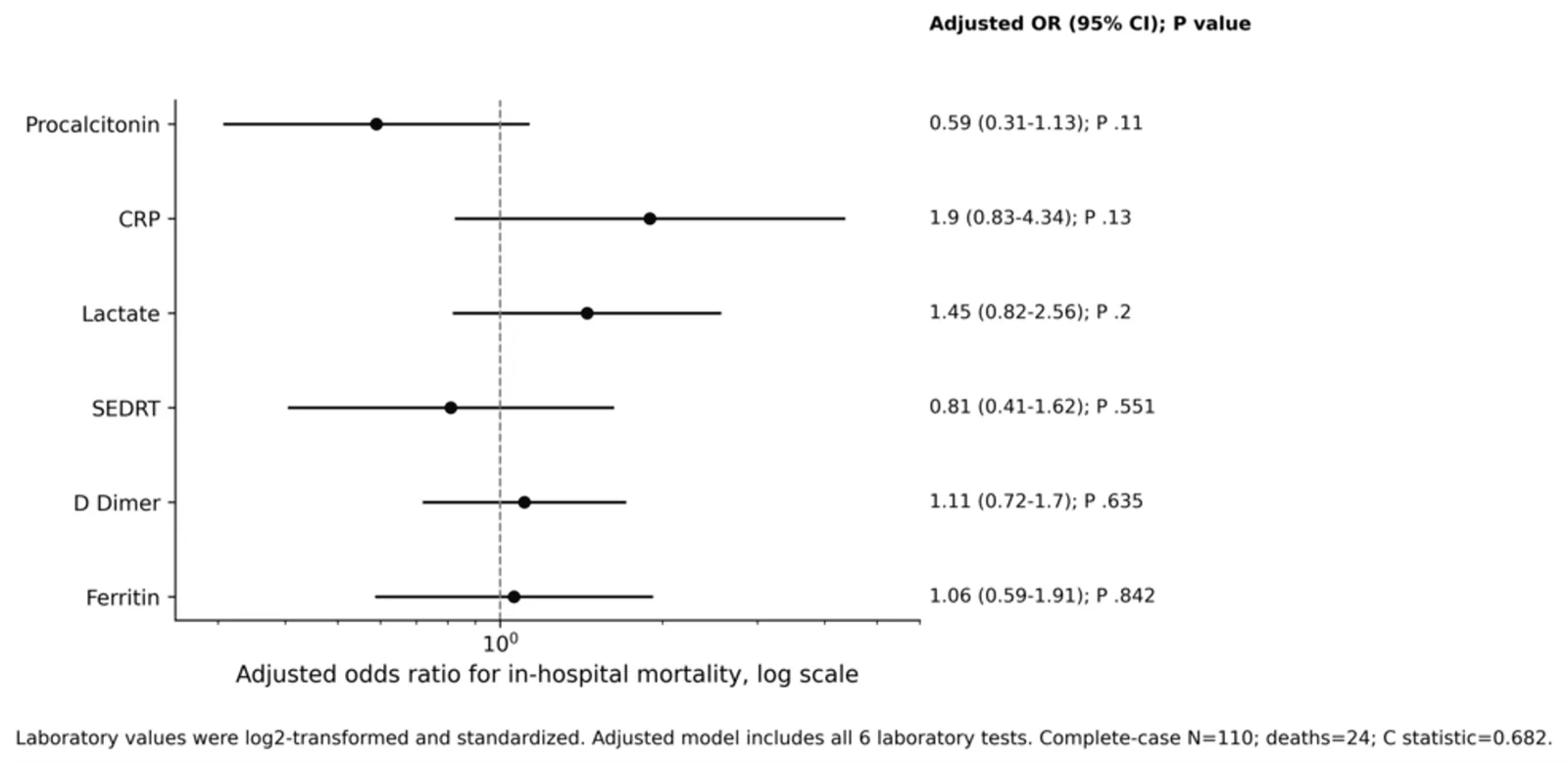
Laboratory predictors of in-hospital mortality. Adjusted odds ratios (ORs) are from the complete-case laboratory-only model; error bars indicate 95% CIs. SEDRT: sedimentation rate

Higher D-dimer levels were associated with increased in-hospital mortality. The point-biserial correlation between log2-transformed D-dimer and mortality was 0.189 (P < 0.001). Mortality increased stepwise across D-dimer quartiles, from 7.9% in the lowest quartile to 23.2% in the highest quartile (Figure 2).

**Figure 2:**
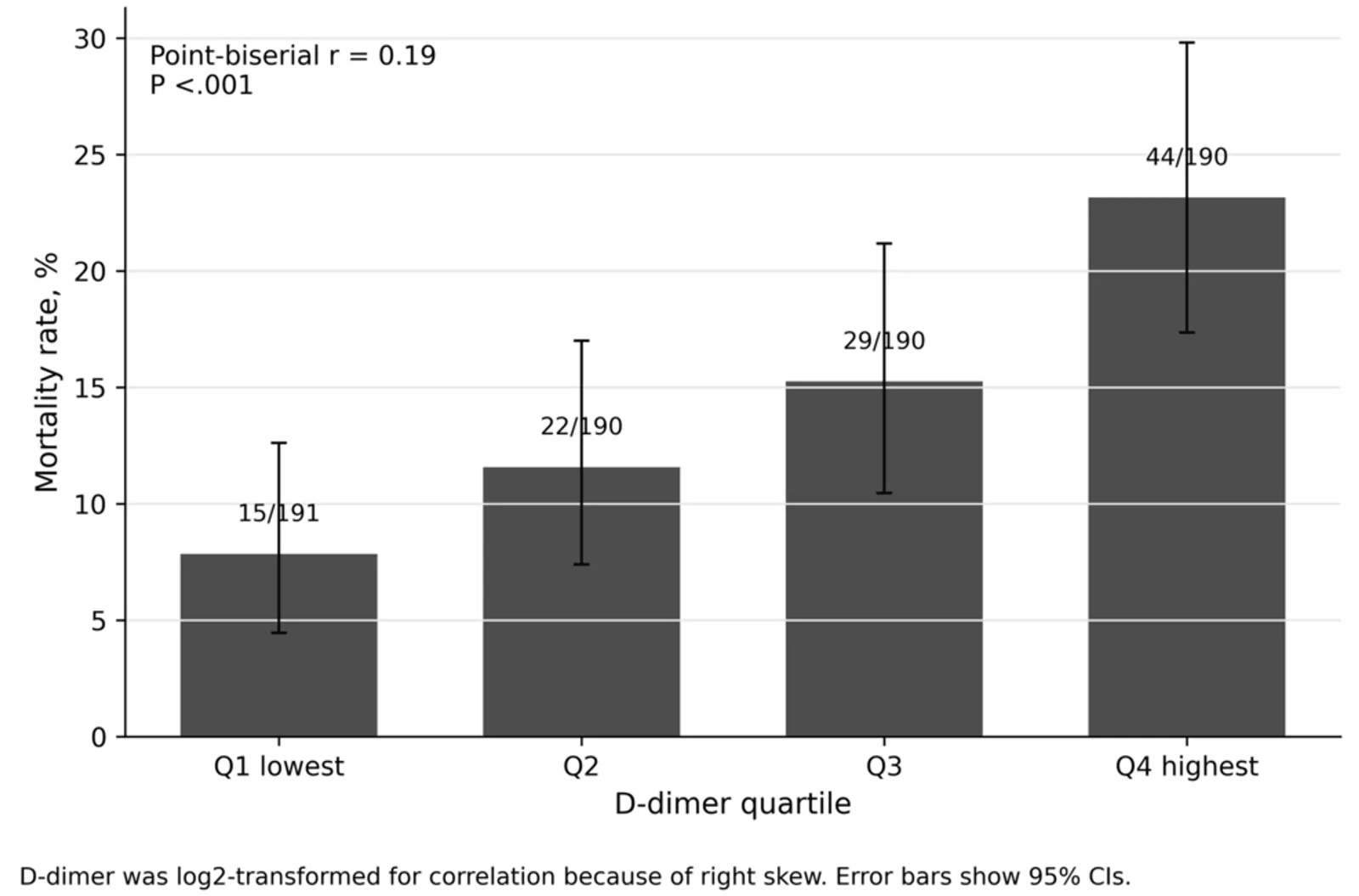
Mortality rate by D-dimer quartile. Error bars show 95% CIs. Log2-transformed D-dimer correlation with mortality was r = 0.189, P < 0.001. This shows a statistically significant positive correlation between D-dimer and mortality. In practical terms, patients with higher D-dimer values had higher mortality rates, although the correlation magnitude was modest. Mortality increased across D-dimer quartiles: Q1 lowest: 15/191 deaths, 7.9%; Q2: 22/190 deaths, 11.6%; Q3: 29/190 deaths, 15.3%; Q4 highest: 44/190 deaths, 23.2%.

In the multivariable clinical mortality model, the strongest independent predictors were mechanical ventilation, older age, and ICU admission. The model had strong discrimination, with a C statistic of 0.913 (Figure 3).

**Figure 3:**
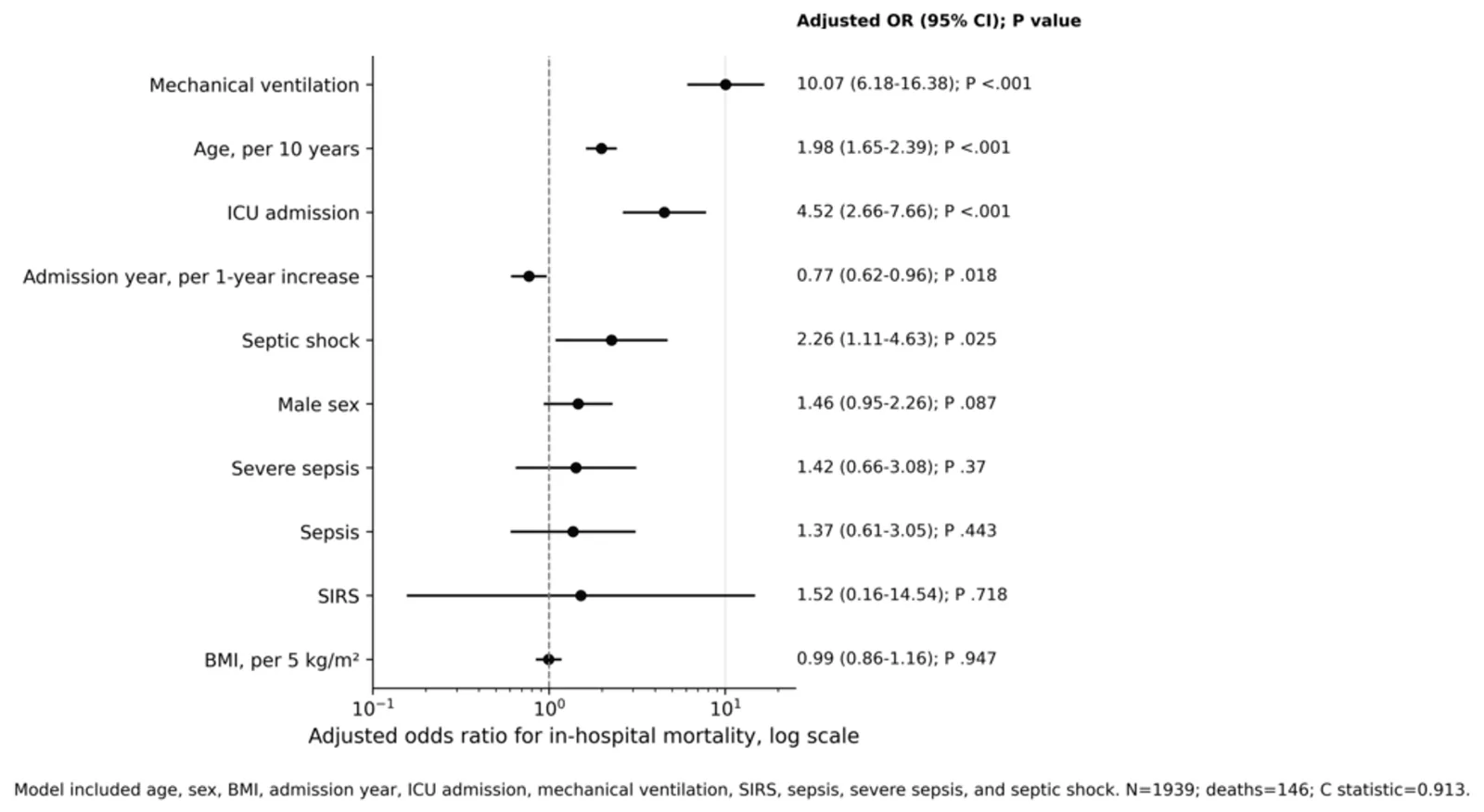
Multivariable clinical predictors of in-hospital mortality. Odds ratios (ORs) are adjusted for all variables shown; error bars indicate 95% CIs. SIRS: systemic inflammatory response syndrome

Among 1,949 patients included in the multivariable analysis, 151 (7.7%) died during hospitalization. In the multivariable logistic regression model adjusted for medication use, age, and sex, receipt of systemic corticosteroids was associated with higher odds of in-hospital mortality (adjusted odds ratio (aOR) 6.70; 95% CI 4.00-11.22; P < 0.001). Receipt of tocilizumab was also associated with higher mortality (aOR 3.87; 95% CI 2.06-7.26; P < 0.001). In contrast, neither remdesivir (aOR 0.90; 95% CI 0.62-1.32; P = 0.60) nor convalescent plasma (aOR 1.63; 95% CI 0.71-3.73; P = 0.25) was significantly associated with mortality. Each one-year increase in age was associated with a 4% increase in the odds of mortality (aOR 1.04; 95% CI 1.03-1.05; P < 0.001), and male patients had higher mortality odds than female patients (aOR 1.58; 95% CI 1.10-2.27; P = 0.01). The model demonstrated moderate-to-good discrimination, with an in-sample area under the receiver operating characteristic curve of 0.785 (Figure 4).

**Figure 4:**
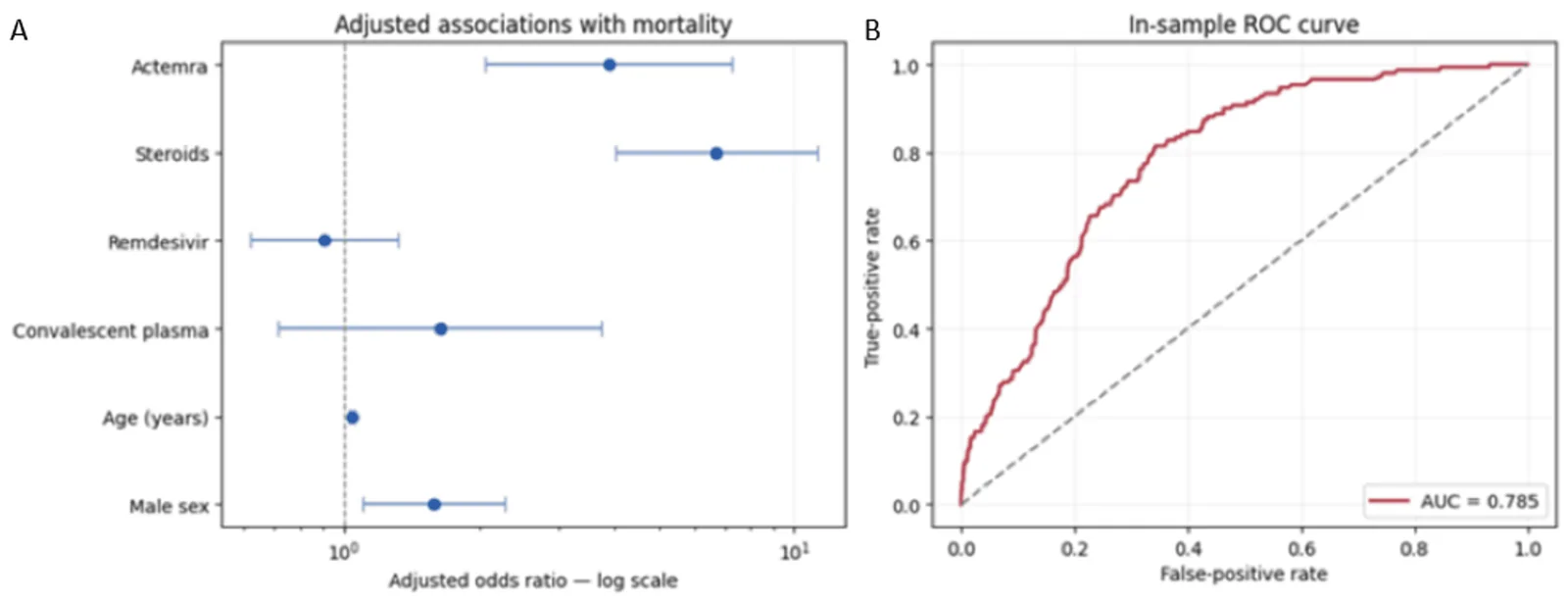
(A, B) A multivariable model showing the association of medication, age, and sex with odds of mortality. ROC: receiver operating characteristic; AUC: area under the curve

Admissions differed by year, increasing from 280 admissions in 2020 to 769 in 2022 and decreasing to 404 in 2023. Mortality was highest in 2021 at 13.9% and was lower in 2022 and 2023, at 4.8% and 5.2%, respectively (Table 3).

**Table 3:** Admissions, mortality, and length of stay by admission year. Values are No. (%) unless otherwise indicated. Length of stay is reported as mean (SD), in days.

Characteristic	Overall	2020	2021	2022	2023	P-value
Admissions, No. (%)	1,949 (100.0)	280 (14.4)	496 (25.4)	769 (39.5)	404 (20.7)	<0.001
Mortality, No. (%)	148 (7.6)	21 (7.5)	69 (13.9)	37 (4.8)	21 (5.2)	<0.001
Length of stay, mean (SD) (d)	9.2 (33.4)	8.9 (8.7)	9.8 (19.6)	6.8 (20.5)	12.9 (64.4)	0.062

In linear regression of outcomes by admission year, mortality decreased by 2.25 percentage points per year, with a 95% CI from -3.47 to -1.03 percentage points and P < 0.001. Length of stay did not demonstrate a statistically significant linear trend, with an estimated annual increase of 0.72 days and P = 0.398 (Table 4 and Figure 5).

**Table 4:** Linear regression of mortality and length of stay by admission year. Mortality was modeled using a linear probability model. Length of stay was modeled using linear regression. Annual changes are shown with 95% CIs.

Outcome	Model	N	Annual change (95% CI)	P-value
Mortality	Linear probability model	1,949	-2.25 percentage points (-3.47 to -1.03)	<0.001
Length of stay	Linear regression	1,644	0.72 days (-0.94 to 2.38)	0.398

**Figure 5:**
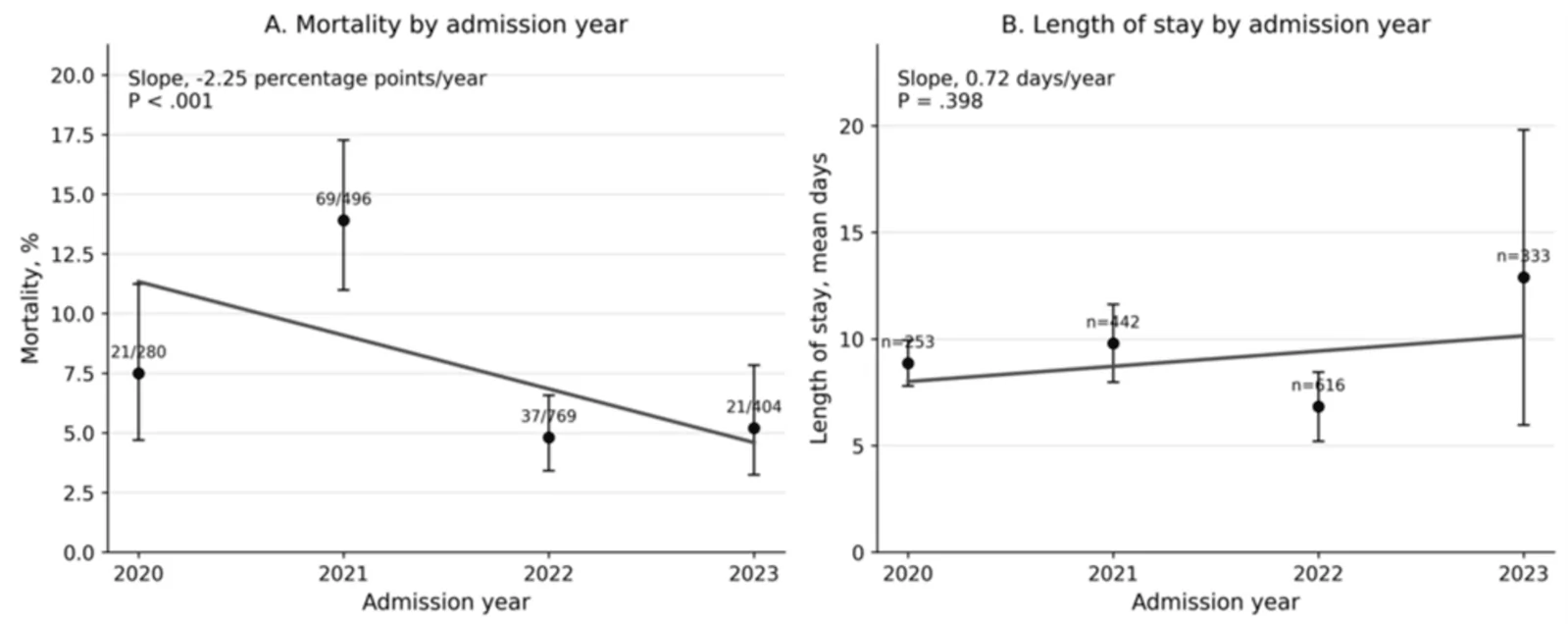
(A, B) Linear regression of mortality and length of stay by admission year. Markers show annual estimates; error bars show 95% CIs.

## Discussion

Laboratory predictors of mortality

Univariable laboratory predictors in the models of inpatient mortality were significant for CRP, lactic acid, ferritin, and D-dimer. Log transformation of D-dimer strengthens the statistically significant positive correlation with mortality. In practical terms, patients with higher D-dimer values had higher mortality rates. In the complete-case adjusted model including all six laboratory tests, no individual laboratory test remained statistically significant. Due to the retrospective design, not all patients had complete lab results. The reduced sample size reflects high missingness or blanks in data fields, which may account for the case-adjusted model demonstrating that no individual lab test was significant.

Clinical predictors of mortality

This retrospective study identified the strongest predictors of in-hospital COVID-19 mortality, including age, septic shock, and mechanical ventilation. Older age was independently associated with increased mortality, with the odds of death nearly doubling for every decade of life (aOR 1.98, 95% CI 1.65-2.39, P < 0.001). This finding is consistent with prior literature. Septic shock and mechanical ventilation are clinical indicators of disease severity. In this study, both are independently associated with mortality. Septic shock was associated with a twofold increase in mortality (aOR 2.26, 95% CI 1.11-4.63, P = 0.025), whereas patients needing mechanical ventilation had a 10-fold increase in the odds of death (aOR 10.07, 95% CI 6.18-16.38, P < 0.001).

Treatment evolution and time

COVID-19 treatment strategies evolved substantially throughout the pandemic. Early management was extrapolated from treatments used for other viral respiratory infections or experimental treatments. As time progressed, treatments were refined into evidence-based interventions such as dexamethasone, remdesivir, proning, and, of course, vaccination. COVID-19 treatment interventions in this study represent physicians' discretion and reflect the community standard of care for that time. In this study, we were not able to account for vaccine status and proning interventions. Systemic steroids were the most frequently used medication (1,014, 51.7%). We hypothesize that steroids were used more frequently than remdesivir due to the guidelines' initial recommendations for antiviral treatment within seven days. It is possible that patients with severe illness at the time of admission were outside of this treatment window. Our multivariable logistic regression model demonstrated a higher association of inpatient mortality in patients treated with systemic corticosteroids (aOR 6.70; 95% CI 4.00-11.22; P < 0.001) and tocilizumab (aOR 3.87; 95% CI 2.06-7.26; P < 0.001). In contrast, neither remdesivir (aOR 0.90; 95% CI 0.62-1.32; P = 0.60) nor convalescent plasma (aOR 1.63; 95% CI 0.71-3.73; P = 0.25) was significantly associated with mortality. Our findings on COVID-19 treatments represent adjusted associations. Furthermore, increased mortality with steroids and tocilizumab is reflective of disease severity and illness severity. These are not causal treatment effects because medication receipt at that time was related to COVID-19 disease severity and confounding by indication for use.

While numerous studies have evaluated the efficacy of single treatments to prevent hospitalization and mortality, no studies capture the real-world cumulative effects of these treatment advances over time. To address this gap, this study conducted a time-lapse analysis of the interval between admission and mortality. The later admission year was independently associated with lower mortality (aOR 0.77 per year, 95% CI 0.62-0.96, P = 0.018). This suggests a 23% reduction in the odds of death with each successive year. We hypothesize that this observed improvement reflects combined advances in clinical management, provider experience, and population immunity over time. Although causality cannot be established from this analysis, it supports an improvement in outcomes over time. These results are at risk for residual confounding, as we are not able to control and account for all treatments during the pandemic that would improve mortality over time.

Limitations

Several limitations should be acknowledged. First, this is a single-center retrospective study at a community hospital, which limits generalizability to other settings and populations [28,29]. Second, vaccination status was not available, precluding assessment of its contribution to the observed mortality decline-a factor that has been shown to reduce in-hospital mortality by approximately 50% in large cohort studies [30]. Third, laboratory analyses were substantially limited by missing data, particularly for ferritin, D-dimer, and SEDRT, reducing the complete-case adjusted model to only 110 patients and likely contributing to the loss of statistical significance for individual biomarkers. Fourth, the use of ICD-10 codes to identify COVID-19 admissions may introduce misclassification, particularly in later pandemic years when incidental SARS-CoV-2 positivity became more common. We correlated these with positive COVID-19 PCR testing to reduce this risk. It is possible that later admissions could have contributed to the declining proportion with a COVID-19 diagnosis code (95.7% in 2020 to 76.5% in 2023), suggesting that some later admissions may have been primarily for non-COVID-19 conditions. Fifth, the study could not account for outpatient treatments (e.g., nirmatrelvir-ritonavir and monoclonal antibodies) that may have prevented hospitalization of higher-risk patients, thereby altering the case-mix of admitted patients over time.

## Conclusions

This study provides a real-world, community hospital perspective on inpatient outcomes throughout the COVID-19 pandemic. Our findings reinforce the prognostic capabilities of laboratory and clinical markers. This includes D-dimer as the strongest laboratory predictor of disease severity and mortality, while the need for mechanical ventilation remains the strongest clinical indicator of severe illness and is associated with the highest risk of inpatient death. Importantly, we observed a progressive improvement in survival over time. These findings likely reflect the combined effects of viral evolution, increasing population immunity, and advances in clinical management with integration of evidence-based therapeutics. Collectively, our results highlight the substantial improvements in inpatient outcomes over the course of the pandemic while reaffirming the prognostic value of established markers of disease severity in hospitalized patients.
